# Glucocorticoid Therapy in Acute Lymphoblastic Leukemia: Navigating Short-Term and Long-Term Effects and Optimal Regimen Selection

**DOI:** 10.1007/s11899-024-00735-w

**Published:** 2024-06-13

**Authors:** Hoda Pourhassan, Lindsey Murphy, Ibrahim Aldoss

**Affiliations:** 1 Department of Hematology and Hematopoietic Cell Transplantation, City of Hope National Medical Center, Duarte, California USA; 2 Department of Pediatrics, City of Hope National Medical Center, Duarte, California USA

**Keywords:** Glucocorticoid, Acute Lymphoblastic Leukemia, Resistance, Regimen

## Abstract

**Purpose of Review:**

Glucocorticoids are a mainstay in acute lymphoblastic leukemia treatment and lack of early response is predictive for overall disease prognosis. Given the vital position of glucocorticoids and well known long and short-term side effects associated with differing glucocorticoids, we aim to highlight the wide breadth of historical and more contemporary data to describe the current landscape of glucocorticoid use in this arena.

**Recent Findings:**

Emerging studies aim to overcome issues such as steroid resistance and to optimize the antileukemic effects of glucocorticoids while aiming to mitigate the risks and side effects associated with their exposure.

**Summary:**

Glucocorticoids have and likely always will be a fundamental component of acute lymphoblastic leukemia treatment and understanding how to navigate short- and long-term effects and how to optimize regimens is at the heart of continued treatment success.

## Introduction

The history of glucocorticoid (GC) use in acute lymphoblastic leukemia (ALL) is long standing and synthetic GCs are noted as one of the first antileukemic components in ALL regimens. The first studies concentrated on comparisons and evaluation of dose response and differences in efficacy, side effect profiles and overall outcomes. As GCs continued to be implemented over decades of use, closer inspection brought to light further complexities including steroid resistance leading to *in vivo* and *in vitro* analyses to better understand these mechanisms and the pathways involved in the hopes for targeted therapies to both overcome this resistance and enhance GC efficacy. This review will serve as a biography of sorts to describe the history and progression of GCs use in ALL and the future we may be able to anticipate in their continued and steadfast use.

## Glucocorticoid Mechanism of Action in Acute Lymphoblastic Leukemia

Glucocorticoids have been an integral and mainstay component of chemotherapy regimens in ALL for decades and poor initial response to GCs is a predictor for treatment failure [1–3]. The mechanisms involved in the efficacy of GCs include multiple biological pathways mediated by the interaction of the GCs and their glucocorticoid receptor (GCR) which can act as a transcriptional activator or repressor. This is achieved through direct binding to DNA or interaction with transcription factors resulting in the ability to induce cell cycle arrest, inhibit cell growth, and mediate apoptotic pathways that ultimately lead to cellular death [1].

## History of Dexamethasone Vs Prednisone in ALL

Due to their lympholytic properties, synthetic GCs including prednisone and dexamethasone have been historically employed as anti-inflammatory and immunosuppressive agents. GGs were among the first drugs which were used for ALL treatment and have been utilized in combination with chemotherapy for decades [4, 5]. Both compounds are synthetic analogs of endogenous cortisol but differ in their molecular structure and in their pharmacokinetic profile. Dexamethasone has greater cytotoxic effect and *in vivo* factors that may contribute to its greater efficacy including a lower protein-bound fraction and longer half-life in both plasma and the cerebral spinal fluid (CSF), leading to better CSF penetration, higher CSF concentrations and reduced rates of CNS relapse observed during induction therapy [6–8].

Early trials comparing these synthetic GCs initially concentrated on evaluating dose comparisons and response and demonstrated that the dose of dexamethasone was positively associated with the degree of bone marrow response [4]. Higher dose dexamethasone treatment abrogated the effect of relative drug insensitivity and of low GCR expression on leukemia blast cells [4]. *In vitro* studies examining antileukemic potency describe a 16-fold higher cytotoxic potential for dexamethasone compared to prednisone [5]. In contrast, several randomized trials investigating various prednisone/dexamethasone ratios lead to consensus estimation of a biologically equipotent ratio of 6 to 7 of prednisone to dexamethasone [9, 10].

The Children's Cancer Group (CCG)-1922 study randomized 1060 patients with standard risk ALL to receive prednisone (40 mg/m^2^/day) or dexamethasone (6 mg/m^2^/day) during remission-induction, consolidation, and maintenance therapy and demonstrated that patients randomized to the dexamethasone arm had higher event-free survival (EFS) (85% vs. 77%; *p*=0.002) and a lower 6-year incidence of isolated CNS relapse (3.7% vs. 7.1%; *p*=0.01) [11••]. Similarly, the Medical Research Council (MRC) ALL 97/99 study randomized 1603 children with standard- and high-risk ALL and those who received dexamethasone (6.5 mg/m^2^/day) rather than prednisolone (40 mg/m^2^/day) during the induction, consolidation, and continuation phases had half the risk of isolated CNS relapse (2.5% vs. 5%; *p*=0.0007) and 5-year EFS was significantly greater with dexamethasone (84.2% vs. 75.6%; *p*=0.007) leading to early closure of randomization given this observed superiority [12••].

Higher EFS is even observed when dexamethasone is used only after induction therapy and can abrogate the adversity of high-risk disease as observed in the Dana Farber Cancer Institute (DFCI) ALL Consortium Protocol 00-01 study of 408 patients randomized to prednisone or dexamethasone every 3 weeks during intensification and continuation therapy, after prednisone-based remission-induction therapy [13••]. Standard-risk patients were randomized to receive prednisone 40 mg/m^2^/day or dexamethasone 6 mg/m^2^/day, while the high-risk group were randomized to receive higher doses (prednisone 120 mg/m^2^/day and dexamethasone 18 mg/m^2^/day) during intensification therapy. The 5-year EFS estimate was 90% in the dexamethasone arm and 81% in the prednisone arm (*p*=0.01) and dexamethasone has proven superiority among patients with high-risk ALL (5-year EFS, 91% vs. 78%, *p*=0.01) compared to standard-risk patients (89% vs. 84%, *p*=0.01). Lastly, in the collaborative clinical trial AIEOP-BFM ALL 2000 conducted by the Associazione Italiana di Ematologia e Oncologia Pediatrica (AIEOP) and Berlin-Frankfurt-Münster (BFM) [14••] evaluating 3655 patients following a 7-day prednisone prophase, higher doses of prednisone (60 mg/m^2^) and dexamethasone (10 mg/m^2^) during induction therapy were evaluated and demonstrated 6-year EFS was greater with dexamethasone versus prednisone (84.1% vs. 79.1%; *p*=0.0083). 6-year cumulative risk of relapse was significantly different (11% for dexamethasone vs. 18% for prednisone; *p*<0.001) and the difference was observed for both isolated bone marrow relapse (8% vs. 12%) and CNS relapse (2% vs. 4%). While the cumulative risk of relapse was reduced by dexamethasone in both ALL phenotypes, it was most pronounced among patients with T-ALL (6% vs. 20%; *p*=0.003) and in those with *ETV6-RUNX1* (4% vs. 13%; *p*<0.001) who had a good response to the prednisone prophase.

The AIEOP-BFM study also evaluated outcomes including the prognostic impact of minimal residual disease (MRD), EFS and overall survival (OS) with use of dexamethasone versus prednisone in induction phase ALL therapy [14••, 15–17]. The use of dexamethasone (10 mg/m2/day) in place of prednisone (60 mg/m2/day) resulted in evident differences in outcomes. 5-year cumulative incidence of relapse (10.8% in the dexamethasone study arm vs. 15.6% with prednisone, *p*=<0.0001), and the largest impact was observed on extramedullary relapses with 5-year EFS rates of 83.9% for dexamethasone and 80.8% for prednisone (*p*=0.024). the benefit of dexamethasone was partially offset by significantly higher induction-related death rates where life-threatening events in the dexamethasone arm included bacterial and fungal infections, as well as neurologic and gastrointestinal complications. At the interim analysis, adolescent patients appeared to be at the highest risk for these complications and as such, 4 years into the trial, randomization for patients 10 years of age and older was stopped. There was no difference in 5-year OS for either GC, however, there was a significant OS benefit from dexamethasone vs prednisone (91.4% vs 82.6%, *p*=0.036) in patients with T-cell ALL who had a good response to the prednisone pre-phase, as well as better EFS and relapse reduction.

## Glucocorticoid Resistance in ALL

The considerable link between primary GC resistance and poor prognosis and outcomes in ALL underscores the significance of GC therapy [18–20]. Poor prednisone response is defined as the presence of ≥1.0 × 10^9^ blasts/L in the peripheral blood on day eight of therapy and predicts significantly higher risk for relapse and worse outcomes and as such, GC resistance identifies a high-risk population with ALL that could benefit from intensifying therapy or an alternative consolidative approach [4, 21–23].

Notably, the precise mechanisms of this resistance have yet to be fully understood. It is postulated that GC treatment may incur selection pressure on leukemic cells leading to acquired genetic changes that weaken a functional steroid response, leading to therapy failure and relapse [24]. Another avenue could be subclones with mutations responsible for GC resistance being already present at the time of diagnosis such that the elimination of GC-sensitive cells causes the resistant subpopulation to become a dominant clone [24].

What is evident is that the GCR plays a significant role in this process. The human GCR is a protein encoded by the *NR3C1* gene comprised of 9 exons [24]. It is widely expressed and binds GC hormones to mediate cellular and tissue-specific effects in development, metabolism, and immune response [25]. As a result of alternate splicing of exon 9, GCRα and GCRβ variants are produced, the latter of which is unable to bind GC. It is such that the GCRβ isoform is thought to contribute to GC resistance in ALL treatment by competing with GCRα at the DNA-binding site and this resistance can be produced by its antagonism towards GCR [25–27]. Other GCR splice variants including GCRγ were discovered to change GCR sensitivity and GCRγ expression has been linked to resistance to dexamethasone treatment in ALL [27, 28]. Other reported mechanisms involving the receptor include lower overall *NR3C1* gene expression which leads to decreased GCR expression and has been linked to poor prognosis and tumor development [23].

Through studies utilizing ALL cell lines *in vitro* and retrospectively evaluating clinical responses, multiple signaling pathways have been implicated in GC resistance during ALL treatment and directly correlated with GC sensitivity. The BCL-2 protein family has been identified as a critical mediator of GC-induced apoptosis and proteasomal degradation of the GCR has also been implicated in resistance to GC treatment [29–31]. GC resistance has been linked to *IKZF1* mutations, which are especially prevalent in Ph-like ALL. In a study of 646 pre-B ALL patients, *IKZF1*-deletions correlated with day 8 prednisone response and were more prevalent in poor prednisone response patients [32]. Activation of the IL-7 signaling pathway plays a crucial role in T- and B-cell development and has been associated with T-ALL resistance to GC treatment [33–35]. Activation of the PI3K/AKT/mTOR signaling cascade prevents the GCR from translocation to the nucleus and it is the activation of AKT1 that may play a role in the development of GC resistance in ALL [36]. The MAPK-ERK pathway which takes part in controlling cellular growth and survival has also been implicated, and this is supported by enhancement of GC sensitivity when GC resistant cell lines were treated with a MAPK inhibitor [37].

## Interventions for Glucocorticoid Resistance

Given the direct correlation between glucocorticoid resistance and outcomes in ALL, there has been significant effort and research dedicated to overcoming this challenge and elucidating methods of enhancing GC therapy. Most of these methods aim to intervene in specific signaling pathways and have been previously discussed in the literature [38].

Cyclic adenosine monophosphate-dependent protein kinase (cAMP-PKA) signaling is one such pathway wherein GCR protein levels are increased to overcome GC resistance and increase GCR levels in T-ALL patient samples [39]. Specific microRNAs (miRNAs) have been implicated by either up or downregulation, leading to alteration in GCR’s nuclear translocation or suppressing its expression and these miRNAs could perhaps be targeted to specifically overcome GC resistance [40, 41]. Overexpression of caspase 1 (CASP1), which cleaves the GCR at its transactivation region can also lead to resistance, and hence, inhibitors of CASP1 have also been implicated for possible future targets [42]. Proteasome inhibitors have also been tried in combination with ALL regimens (i.e. carfilzomib). However, when combined with dexamethasone, mitoxantrone, methotrexate, pegylated L-asparaginase, and vincristine (UKALLR3) during induction therapy, carfilzomib was found to be excessively toxic [43]. Conversely, in another phase 1 study of ten patients with Philadelphia chromosome (Ph) negative ALL undergoing induction, when carfilzomib was combined with hyper-fractionated cyclophosphamide, vincristine, doxorubicin, and dexamethasone (HyperCVAD), the treatment was well tolerated with 90% CR rates following initial cycle and 70% of patients achieving MRD negative remission [44].

In the case of Ph- positive B-cell ALL, where tyrosine kinase inhibitors are now implemented as standard of care, dasatinib has been of particular interest. This is owed to the observed relationship between GC-resistance and B-cell development with activation of downstream PI3K/mTOR and CREB signaling and dasatinib’s dual SRC/ABL inhibitor mechanism which inhibits these pathways. The combination of dasatinib with dexamethasone results in increased cell death *in vitro* and increased survival in an *in vivo* model, suggesting dasatinib may be beneficial in GC resistance in ALL [45].

T-ALL poses another unique space often showing resistance to the first 7 days of GC treatment and with more limited treatment options in the context of relapse, classifying these cases as high-risk disease. This resistance has also correlated with inferior disease-free survival even following high-risk adapted therapy and these patients tend to respond poorly compared to other high-risk cohorts [2, 46–48]. As such, varied combinations have been studied in this space including NOTCH1 inhibitors such as gamma secretase inhibitors (GSIs). GSIs lead to a reduction of the levels of NR3C1 transcriptional repressor and restored GC receptor self-activation, leading to reversal of resistance to GCs [49]. Unfortunately, due to pan-NOTCH signaling inhibition, various GSIs implemented in clinical trials have had limited efficacy due to toxicities observed but this remains an area of active study [50]. IL-7 receptor pathway inhibitors have also been evaluated using different agents such as the JAK1/2 inhibitor ruxolitinib which alters the balance between proapoptotic and antiapoptotic factors [51] and IL-7 receptor specific blockade with monoclonal antibody to attenuate the dexamethasone-induced increase of cell-surface IL-7 receptor and overcome IL-7-induced dexamethasone resistance [52].

## Glucocorticoid Side Effects

GCs are associated with many adverse effects including bone toxicities, infection that can be lethal, hyperglycemia, myopathy and neuropsychological issues and these effects appear to be more pronounced with age, specifically adults and children over the age of 10 in pediatric studies [53] Table 1. It should also be accounted for that these effects can occur years after cessation of therapy making mitigation of these effects even more pertinent.

**Table 1:** Glucocorticoid Side Effects

Side Effect	Risk Factors	Dexamethasone vs Prednisone
Low bone mineral density	• Younger age • Lower weight	Higher incidence with dexamethasone
Osteonecrosis	• Age • ≥10 years • Higher risk in young (<30) vs older adults • Female sex • White race	Higher incidence with dexamethasone and dose dependent in age ≥10 years
Infection and Mortality	• Prolonged and higher doses of glucocorticoid	Higher incidence with dexamethasone especially in patients ≥ 10 years
Hyperglycemia	• • Age (specifically >60 years in adults) • BMI • Trisomy 21 • Concomitant asparaginase type	Variable study results
Myopathy	• Younger age • Male Sex	Higher incidence with dexamethasone
Neuropsychological	• Older age at diagnosis with dexamethasone Younger age at diagnosis with prednisone • Female sex	Higher incidence with dexamethasone

### Bone Toxicities

The infrastructure of bone is a dichotomy of resorption and formation, mediated by osteoclasts and osteoblasts, respectively, necessitating a fine balance between the two. GCs affect the function of mesenchymal stem cells, osteoblasts, osteocytes and osteoclasts by interfering with various pathways resulting in increased bone resorption rather than formation, eventual osteoporosis and increased fracture rates [54]. The effect of GC treatment is described as biphasic and characterized by early bone resorption followed by prolonged impairment of bone formation [55].

Patients with ALL have low bone mineral density (BMD) values prior to even initiating any antineoplastic therapy, and this is thought to be attributed to infiltration of the bones by malignant cells in addition to other autocrine factors [56–59]. Risk factors for lower BMDs at time of ALL diagnosis include younger age and lower weight [55]. A prospective analysis of ALL patients revealed that BMD deficits were already present in the first month of induction chemotherapy despite normal range BMD values at the time of diagnosis [60]. Furthermore, bone loss has been documented after the cessation of chemotherapy in cohorts of adult survivors of treated pediatric ALL [61, 62].

BMD deficits and higher rate of fractures have been associated with the administration of GCs in ALL treatment regimens and this has been correlated with cumulative GC dose and notably, these effects were observed with higher incidence in dexamethasone when compared to prednisone [56, 57, 63–65]. Furthermore, it has also been reported in two cohorts of childhood ALL survivors that increased GC dose during chemotherapy negatively impacted femoral neck bone mass [60, 64, 65].

A predominance of published data indicates an effect of dexamethasone exposure on the incidence of osteonecrosis (ON) with associated risk factors of cumulative dose of dexamethasone, age ≥10 years, female sex, and white race/ethnicity [66–69]. The CCG reported an overall incidence of ON of 14.2% in 893 patients ≥10 years of age versus 0.9% (*p*=0.0001) in 516 younger patients [66]. The effect of age on ON rates has been well described. In the ALL-BFM-95 study of 1951 patients, the incidence of ON was 8.9% in patients ≥10 years of age and 0.2% in younger patients (*p*=0.001) [70]. In the DFCI 00-01 study, 5-year cumulative incidence of ON was significantly higher at 23% with dexamethasone treatment vs. 4.7% (*p*=0.02) in those treated with prednisone among patients 10–18 years of age, whereas no difference was seen among patients 1–10 years of age [13]. Additionally, it needs to be considered that the occurrence of ON extends to well beyond treatment cessation as evidenced by Kadan-Lottick et al who illustrated a 20-year cumulative incidence of ON 6.2 times of that reported by their siblings among 9261 long-term cancer survivors in the Childhood Cancer Survivor Study and relative risk was 2.7, with higher incidence in those treated with dexamethasone over prednisone [71]. The overall high incidence of aseptic ON in the adolescent patient subgroup of AIEOP-BFM ALL 2000 [17] was comparable with the incidence reported in other ALL trials, however, there was not an excess of aseptic ON observed in the patients treated with dexamethasone.

Bone toxicities have been overall less studied in adults; however, younger adults have been described to be at higher risk than older adults regardless of implementing pediatric inspired or traditional adult treatment protocols [72–74]. ON has been described amongst the adult population and in general some studies have shown higher incidence with female sex and a possible protective effect of African ancestry [66, 68, 75].

Orthopedic toxicities among adolescents and young adults were reviewed and reported amongst 367 patients aged 15-50 years old treated on sequential DFCI ALL Consortium trials [72]. 5-year cumulative incidence of ON and bone fracture was reported at 17% (95% CI, 13-22) and 12% (95% CI, 8-15), respectively. Amongst the group, patients less than 30 years were at higher risk of ON with 5-year cumulative incidence of 21% vs 8%; *p*=0.004. Additionally, there was thought to be a pharmacokinetic drug interaction between pegaspargase and dexamethasone leading to increased dexamethasone exposure and therefore impending orthopedic toxicity given that patients treated more recently on pegaspargase-based protocols were significantly more likely to be diagnosed with ON compared with those treated on earlier trials with native *Escherichia coli* asparaginase (5-year cumulative incidence, 24% vs 5%; P < .001).

### Infection and Mortality

Prolonged exposure to high-dose dexamethasone in conjunction with myelosuppressive therapy can cause severe infections and result in mortality. In the DFCI 91-01P protocol, 16 of 38 children evaluated had documented sepsis when dexamethasone (6 mg/m2) was substituted for prednisone (40 mg/m2) [76], 4 of these patients died as a result. In the ALL AIEOP/BFM 2000 study, the use of 10 mg/m2 of dexamethasone was significantly associated with death during induction, caused largely by severe bacterial and fungal infections [14••]. The cumulative incidence of death when dexamethasone is used instead of prednisone was especially high in patients ≥ 10 years of age (4.5% vs. 2.4%, *p*=0.13) resulting in a halt of randomization for patients ≥ 10 years of age and utilization of prednisone. The DFCI 00-01 protocol showed a significantly higher incidence of infection with dexamethasone (18.8%) verses prednisone (10.6%); *p*=0.03, even though randomization was performed after remission-induction therapy [13••].

### Hyperglycemia

Corticosteroid-induced hyperglycemia is a well-known side effect profile of GCs in general and is often in need of mitigation during ALL treatment protocols given the propensity to lead to other complications such as increased risk of bacterial, viral and fungal infections [77]. The mechanism by which GCs induce hyperglycemia is thought to be by affecting the pancreatic beta cell and decreasing insulin synthesis, increasing insulin resistance, stimulating gluconeogenesis and lipolysis, and enhancing counter-regulatory hormones [78, 79].

The prevalence of hyperglycemia amongst pediatric ALL cases is variably reported in studies and over the historical review of pediatric literature, prevalence reported in studies has increased possibly owed to the increased use of dexamethasone over prednisone despite no evidence available to differentiate hyperglycemic capacity between the two GCs. As an example, in the CCG-11922 study, patients who received dexamethasone had a significantly higher incidence of reversible grade 3 or 4 hyperglycemia compared to those who received prednisone (5% vs 1.5%; P = .001) [11••]. Prevalence in pediatric literature is wide ranged depending on the study anywhere between 11% to 56% according to literature review [77]. There have been fewer studies of hyperglycemia in adults with ALL, however, the prevalence has been reported to be 37% [77, 80].

Age is the only statistically significant risk factor reported for GC-induced hyperglycemia [81, 82]. In the pediatric population, this has been thought to be owed to puberty, a time in which sex steroids and growth hormone surge, resulting in an increased insulin resistance and therefore altered glucose metabolism [83]. Amongst the adult population, older age has also been correlated with the increased propensity for hyperglycemia induction, specifically age >60 years [80]. There are also potential risk factors which have had variable statistical significance in the literature including BMI (obesity), Trisomy 21 and concomitant asparaginase type used [77].

Hyperglycemia-related complications in ALL are variable. Diabetic ketoacidosis (DKA) and hyperglycemic hyperosmolar state are complications that have been described in adults with ALL and though not extensively studied in the pediatric population, have been reported [84, 85]. Hyperglycemia is significantly associated with a higher risk of infections (bacterial, fungal and viral) as well as febrile neutropenia and there has been a proposed dose response effect between severity of hyperglycemia and the risk for infection [77, 81, 84].

### Myopathy

Proximal myopathy is a complication of corticosteroid therapy and has been reported in other oncological contexts requiring continued GC use from which we can extrapolate [86]. Steroid myopathy incidence has been reviewed amongst ALL patients receiving GC therapy such as in the CCG-1922 study group where patients randomized to dexamethasone had a significantly greater prevalence of reversible grade 1–3 steroid myopathy vs. those who received prednisone (6.3% vs. 1.5%) and grade 3 weakness (4.1% dexamethasone vs. 0.3% for prednisone, *p*=<0.0001) during or immediately after induction therapy [11••]. Amongst this specific cohort, younger age and male sex were risk factors for development of severe weakness. In the MRC ALL 97/99 study, transient myopathy during induction therapy was 2.8% in the dexamethasone arm and 0.5% in the prednisolone arm (*p*=0.001) again reiterating higher toxicity with dexamethasone and occurrence of this complication [12••]. Almost all these patients had lower limb involvement, most frequently of the quadriceps and/or glutei and recovery occurred after induction.

### Neuropsychological Effects

As previously described, given the superior CNS penetration of dexamethasone, its effect on neuropsychological outcomes is significant in terms of quality-of-life. The entity of “steroid psychosis” has been well established and discussed as a general side effect of GC therapy [87, 88]. In the MRC ALL 97/99 study, dexamethasone as compared to prednisone was associated with more frequent abnormal behavior and appeared to manifest more often as depression in females and aggression towards self or others in males. There was also observed overall emotional lability and mood swings in general more pronounced with dexamethasone than prednisone [12••]. There are also some historical studies alluding to possible effects on neurocognitive function and academic performance in ALL patients exposed to GCs but the results for this have overall lead to the conclusion that there is no significant overall difference other than possibly one-third of a standard deviation lower scoring on a test of word reading [89, 90]. The relationship between specific GC and neurocognitive outcomes is possibly influenced by age at diagnosis and sex, with older age at diagnosis and use of dexamethasone being associated with worse IQ, processing speed, spelling, and reading while younger age at diagnosis with prednisone is associated with worse functioning [90]. Female sex has also been associated with worse processing speed for patients who received prednisone, but not for other areas of neurocognitive functioning [90].

## Conclusions and Future Directions

It is clear that GCs have been and will continue to be an absolutely central component to the successful treatment of ALL across the age spectrum. The culmination of data which we continue to rely on has tailored to the selective use of either dexamethasone or prednisone specifically depending on age, and this breaks down beyond just pediatric and adult spaces with selective use of dexamethasone in pediatric patients <10 years of age and prednisone above age 10 based on the cumulative results of the hallmark historic studies reviewed given observed side effect profiles, long and short term effects and of course, differences in outcomes [11••, 12••, 13••, 14••]. In the adult realm, age adapted steroid dosing has been a recent endeavor with substitution of dexamethasone for prednisone in the original C10403 protocol which historically implemented prednisone day 1-28 [91]. Although this modification was instigated given a predominance of CNS relapse observed, hyperglycemia appeared to be the main toxicity that could be attributed to GC, while the remaining side effect profile appeared to be more related to asparaginase. It should be considered that the purpose of the study was not specifically to mitigate or evaluate GC toxicity but gives a glimpse to possible future directions of GC implementation and optimization of efficacy with less toxicity.

**Fig. 1:**
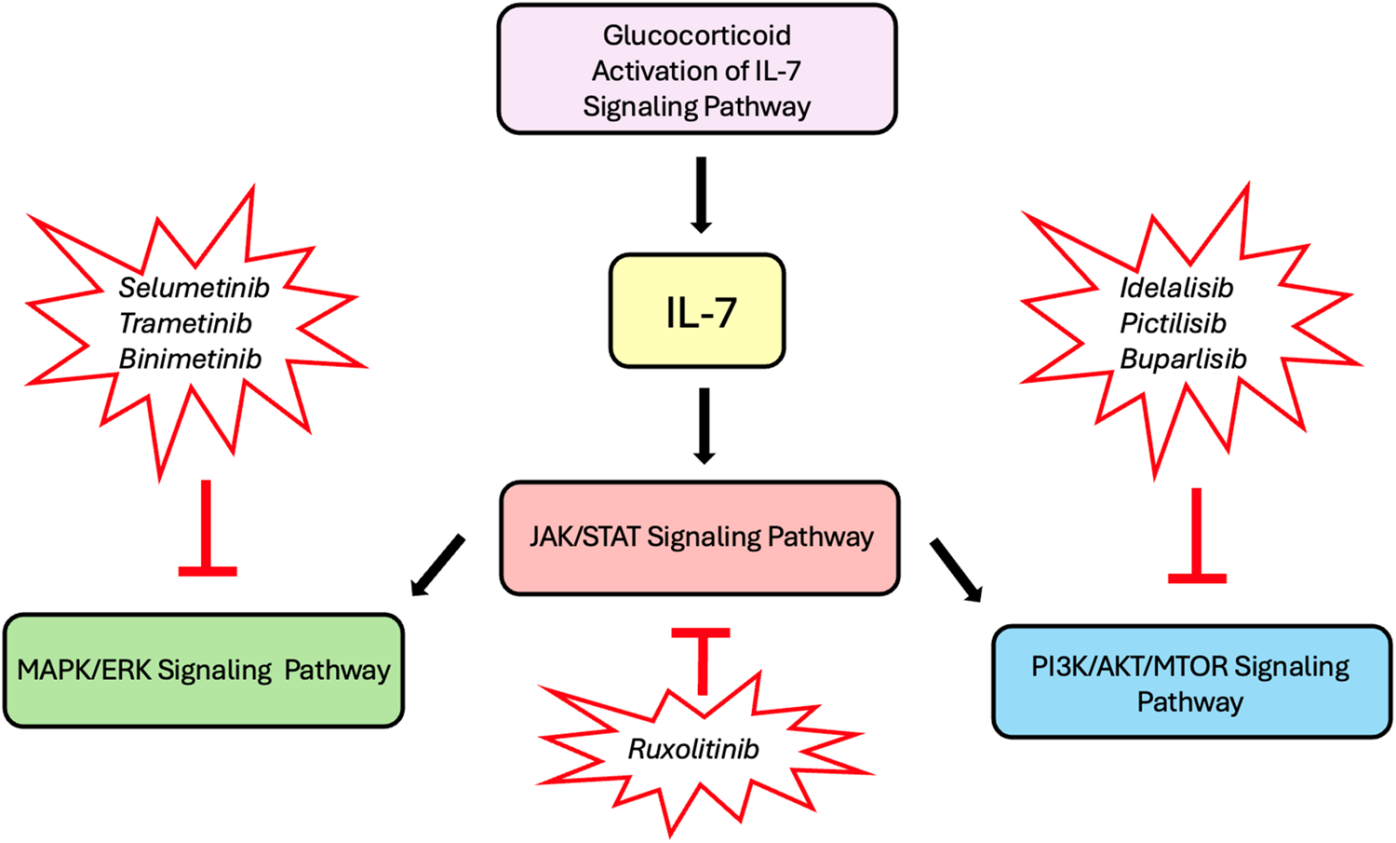
Agents Used in Glucocorticoid Resistance. Signaling pathways including downstream JAK/STAT, PI3K/AKT/mTOR, and MAPK-ERK have been identified in glucocorticoid resistance and have been shown to be induced by activation of the the IL-7 signaling pathway. Agents capable of blocking signaling pathways involved in the ALL glucocorticoid resistance including Idelalisib, Pictilisib and Buparlisib (PI3 kinase inhibition), ruxolitinib (JAK 1/2 inhibition), and Selumetinib, Trametinib and Binimetinib (MAPK inhibition) have been explored

As discussed, steroid resistance in and of itself has been shown to confer poor prognosis and outcomes in ALL (18-20) and future directions of ALL therapy could perhaps concentrate on intensifying and enhancing GC efficacy and overcoming resistance by targeting specific players in the GC/GCR signaling pathways shown to be vital in this respect. As just one example, the BCL-2 protein family has been identified as a critical mediator of GC-induced apoptosis and proteasomal degradation of the GCR has also been implicated in resistance to GC treatment [29–31]. Venetoclax, a BCL-2 inhibitor, has been gaining interest in the treatment armamentarium of ALL given multiple studies demonstrating efficacy across the phenotypic spectrum, including T-cell and early T-cell progenitor (ETP) ALL, often showing resistance to the first 7 days of GC treatment [46–48, 92–98]. Multiple signaling pathways have been identified in GC resistance and have been shown to be induced by activation of the IL-7 signaling pathway, including downstream JAK/STAT, PI3K/AKT/mTOR, and MAPK-ERK signaling pathways [38]. As such, many agents capable of blocking signaling pathways involved in the ALL glucocorticoid resistance have already been explored, including Idelalisib, Pictilisib and Buparlisib (PI3 kinase inhibition), ruxolitinib (JAK 1/2 inhibition), and Selumetinib, Trametinib and Binimetinib (MAPK inhibition) underscoring the significantly fertile ground to implement targeted therapies to allow for continued optimization of GC use in the treatment of ALL which remains to be further explored [99–112] Fig. 1.

The toxicity profile of GCs has been intensely studied in multiple arenas of medical practice given the large breadth of efficacy across disease states and specialties. In the case of ALL, there is no question that despite their short and long-term effects, GCs are here to stay. Obesity is already prevalent in the ALL population and there is association between obesity, insulin resistance, metabolic syndrome and chemotherapy resistance in ALL, all of which are exacerbated by GC use [113, 114]. Obesity is also associated with increased risk of relapse as well as poor survival likely from metabolic effects that promote survival of leukemia cells [115]. Glucagon-like peptide-1 (GLP-1) analogues and other obesity interventions are gaining significant attention in the obesity epidemic [116, 117] and could perhaps also be an added area of exploration for ways to overcome the adverse effects of GC use that is vital to ALL therapy.

## References

[1] Nguyen K, et al. Factors influencing survival after relapse from acute lymphoblastic leukemia: a Children’s Oncology Group study. Leukemia. 2008;22:2142–50. 18818707 10.1038/leu.2008.251 PMC2872117

[2] Dördelmann M, et al. Prednisone response is the strongest predictor of treatment outcome in infant acute lymphoblastic leukemia. Blood. 1999;94(1209–1217):47. 10438708

[3] DeVita VT, Chu E. A history of cancer chemotherapy. Cancer Res. 2008;68:8643–53. 18974103 10.1158/0008-5472.CAN-07-6611

[4] Inaba H, Pui CH. Glucocorticoid use in acute lymphoblastic leukaemia. Lancet Oncol. 2010;11:1096–106. 20947430 10.1016/S1470-2045(10)70114-5 PMC3309707

[5] Kaspers GJ, Veerman AJ, Popp-Snijders C, et al. Comparison of the antileukemic activity in vitro of dexamethasone and prednisolone in childhood acute lymphoblastic leukemia. Med Pediatr Oncol. 1996;27:114–21. 8649318 10.1002/(SICI)1096-911X(199608)27:2<114::AID-MPO8>3.0.CO;2-I

[6] Balis FM, Lester CM, Chrousos GP, Heideman RL, Poplack DG. Differences in cerebrospinal fluid penetration of corticosteroids: possible relationship to the prevention of meningeal leukemia. J Clin Oncol. 1987;5(2):202–7. 3806166 10.1200/JCO.1987.5.2.202

[7] Jones B, Freeman AI, Shuster JJ, et al. Lower incidence of meningeal leukemia when prednisone is replaced by dexamethasone in the treatment of acute lymphocytic leukemia. Med Pediatr Oncol. 1991;19(4):269–75. 2056971 10.1002/mpo.2950190411

[8] Veerman AJ, Hählen K, Kamps WA, et al. High cure rate with a moderately intensive treatment regimen in non-high-risk childhood acute lymphoblastic leukemia. Results of protocol ALL VI from the Dutch Childhood Leukemia Study Group. J Clin Oncol. 1996;14(3):911–8. 8622039 10.1200/JCO.1996.14.3.911

[9] Quddus FF, Leventhal BG, Boyett JM, Pullen DJ, Crist WM, Borowitz MJ. Glucocorticoid receptors in immunological subtypes of childhood acute lymphocytic leukemia cells: a Pediatric Oncology Group Study. Cancer Res. 1985;45:6482–6. 3864532

[10] Tissing WJ, Meijerink JP, den Boer ML, Pieters R. Molecular determinants of glucocorticoid sensitivity and resistance in acute lymphoblastic leukemia. Leukemia. 2003;17:17–25. 12529655 10.1038/sj.leu.2402733

[11••] . Bostrom BC, Sensel MR, Sather HN, et al. Dexamethasone versus prednisone and daily oral versus weekly intravenous mercaptopurine for patients with standard-risk acute lymphoblastic leukemia: a report from the Children's Cancer Group. Blood. 2003;101:3809–17. **Landmark paper highlighting higher EFS and less CNS relapse with dexamethasone.** 12531809 10.1182/blood-2002-08-2454

[12••] . Mitchell CD, Richards SM, Kinsey SE, Lilleyman J, Vora A, Eden TO. Benefit of dexamethasone compared with prednisolone for childhood acute lymphoblastic leukaemia: results of the UK Medical Research Council ALL97 randomized trial. Br J Haematol. 2005;129:734–45. **Landmark paper highlighting superiority of dexamethasone over prednisone resulting in early closure of randomization.** 15952999 10.1111/j.1365-2141.2005.05509.x

[13••] . Vrooman LM, Neuberg DS, Stevenson KE, Supko JG, Sallan SE, Silverman LB. Dexamethasone and individualized asparaginase dosing are each associated with superior event-free survival in childhood acute lymphoblastic leukemia: results from DFCI-ALL Consortium Protocol 00-01. Blood (ASH Annual Meeting Abstracts). 2009;114:321. **Landmark paper demonstrating that higher EFS is even observed when dexamethasone is used only after induction therapy and can abrogate the adversity of high-risk disease.**

[14••] . Schrappe M, Zimmermann M, Moricke A, et al. Dexamethasone in induction can eliminate one third of all relapses in childhood acute lymphoblastic leukemia (ALL): results of an international randomized trial in 3655 Patients (Trial AIEOP-BFM ALL 2000). Blood (ASH Annual Meeting Abstracts). 2008;112:7. **Landmark study of higher dose prednisone vs dexamethasone during induction demonstrating overall reduced relapse risk and improved outcomes with dexamethasone though offset but risk of life-threatening infection, specifically patients aged 10 or older.**

[15] Conter V, Bartram CR, Valsecchi MG, et al. Molecular response to treatment redefines all prognostic factors in children and adolescents with B-cell precursor acute lymphoblastic leukemia: results in 3184 patients of the AIEOP-BFM ALL 2000 study. Blood. 2010;115(16):3206–14. 20154213 10.1182/blood-2009-10-248146

[16] Schrappe M, Valsecchi MG, Bartram CR, et al. Late MRD response determines relapse risk overall and in subsets of childhood T-cell ALL: results of the AIEOP-BFM-ALL 2000 study. Blood. 2011;118(8):2077–84. 21719599 10.1182/blood-2011-03-338707

[17] Möricke A, Zimmermann M, Stanulla M, Biondi A, et al. Dexamethasone vs prednisone in induction treatment of pediatric ALL: results of the randomized trial AIEOP-BFM ALL 2000. Blood. 2016;127(17):2101–12. 26888258 10.1182/blood-2015-09-670729

[18] Reiter A, Schrappe M, Parwaresch R, Henze G, Müller-Weihrich S, Sauter S, et al. Non-Hodgkin's lymphomas of childhood and adolescence: results of a treatment stratified for biologic subtypes and stage–a report of the Berlin-Frankfurt-Münster Group. J Clin Oncol. 1995;13:359–72. 7844597 10.1200/JCO.1995.13.2.359

[19] Beesley AH, Palmer ML, Ford J, Weller RE, Cummings AJ, Freitas JR, et al. Authenticity and drug resistance in a panel of acute lymphoblastic leukaemia cell lines. Br J Cancer. 2006;95:1537–44. 17117183 10.1038/sj.bjc.6603447 PMC2360743

[20] Beesley AH, Weller RE, Senanayake S, Welch M, Kees UR. Receptor mutation is not a common mechanism of naturally occurring glucocorticoid resistance in leukaemia cell lines. Leuk Res. 2009;33:321–5. 18789525 10.1016/j.leukres.2008.08.007

[21] Hunger S. P Glucocorticoid Selection for Pediatric ALL. Blood. 2016;127:2049–51. 27127298 10.1182/blood-2016-02-701664

[22] Borin C, Pieters T, Serafin V, Ntziachristos P. Emerging Epigenetic and Posttranslational Mechanisms Controlling Resistance to Glucocorticoids in Acute Lymphoblastic Leukemia. HemaSphere. 2023;7(7):e916. 37359189 10.1097/HS9.0000000000000916 PMC10289758

[23] Gao J, Liu WJ. Prognostic Value of the Response to Prednisone for Children with Acute Lymphoblastic Leukemia: A Meta-Analysis. Eur Rev Med Pharmacol Sci. 2018;22:7858–66. 30536331 10.26355/eurrev_201811_16411

[24] Van der Zwet JCG, Smits W, Buijs-Gladdines JGCAM, Pieters R, Meijerink JPP. Recurrent NR3C1 Aberrations at First Diagnosis Relate to Steroid Resistance in Pediatric T-Cell Acute Lymphoblastic Leukemia Patients. HemaSphere. 2020;5:e513. 33364552 10.1097/HS9.0000000000000513 PMC7755520

[25] Caratti G, Matthews L, Poolman T, Kershaw S, Baxter M, Ray D. Glucocorticoid Receptor Function in Health and Disease. Clin. Endocrinol. 2015;83:441–448. 26. 10.1111/cen.12728 25627931

[26] Nick ZLU, Cidlowski JA. The Origin and Functions of Multiple Human Glucocorticoid Receptor Isoforms. Ann N Y Acad Sci. 2004;1024:102–23. 15265776 10.1196/annals.1321.008

[27] Lu NZ, Cidlowski JA. Translational Regulatory Mechanisms Generate N-Terminal Glucocorticoid Receptor Isoforms with Unique Transcriptional Target Genes. Mol Cell. 2005;18:331–42. 15866175 10.1016/j.molcel.2005.03.025

[28] Cain DW, Cidlowski JA. Specificity and Sensitivity of Glucocorticoid Signaling in Health and Disease. Best Pract Res Clin Endocrinol Metab. 2015;29:545–56. 26303082 10.1016/j.beem.2015.04.007 PMC4549805

[29] Beger C, Gerdes K, Lauten M, Tissing WJE, Fernandez-Munoz I, Schrappe M, Welte K. Expression and Structural Analysis of Glucocorticoid Receptor Isoform Gamma in Human Leukaemia Cells Using an Isoform-Specific Real-Time Polymerase Chain Reaction Approach. Br J Haematol. 2003;122:245–52. 12846893 10.1046/j.1365-2141.2003.04426.x

[30] Jing D, Bhadri VA, Beck D, Thoms JAI, Yakob NA, Wong JWH, Knezevic K, Pimanda JE, Lock RB. Opposing Regulation of BIM and BCL2 Controls Glucocorticoid-Induced Apoptosis of Pediatric Acute Lymphoblastic Leukemia Cells. Blood. 2015;125:273–83. 25336632 10.1182/blood-2014-05-576470

[31] Malyukova A, Brown S, Papa R, O’Brien R, Giles J, Trahair TN, Dalla PL, Sutton R, Liu T, Haber M, et al. FBXW7 Regulates Glucocorticoid Response in T-Cell Acute Lymphoblastic Leukaemia by Targeting the Glucocorticoid Receptor for Degradation. Leukemia. 2013;27:1053–62. 23228967 10.1038/leu.2012.361

[32] Marke R, Havinga J, Cloos J, Demkes M, Poelmans G, Yuniati L, van Ingen Schenau D, Sonneveld E, Waanders E, Pieters R, et al. Tumor Suppressor IKZF1 Mediates Glucocorticoid Resistance in B-Cell Precursor Acute Lymphoblastic Leukemia. Leukemia. 2015;30:1599–603. 26713593 10.1038/leu.2015.359

[33] Wilkinson L, Verhoog NJD, Louw A. Disease- and Treatment-Associated Acquired Glucocorticoid Resistance. Endocr Connect. 2018;7:R328–49. 30352419 10.1530/EC-18-0421 PMC6280593

[34] Li Y, Buijs-Gladdines JGCAM, Canté-Barrett K, Stubbs AP, Vroegindeweij EM, Smits WK, van Marion R, Dinjens WNM, Horstmann M, Kuiper RP, et al. IL-7 Receptor Mutations and Steroid Resistance in Pediatric T Cell Acute Lymphoblastic Leukemia: A Genome Sequencing Study. PLoS Med. 2016;13:e1002200. 27997540 10.1371/journal.pmed.1002200 PMC5172551

[35] Delgado-Martin C, Meyer LK, Huang BJ, Shimano KA, Zinter MS, Nguyen JV, Smith GA, Taunton J, Winter SS, Roderick JR, et al. JAK/STAT Pathway Inhibition Overcomes IL7-Induced Glucocorticoid Resistance in a Subset of Human T-Cell Acute Lymphoblastic Leukemias. Leukemia. 2017;31:2568–76. 28484265 10.1038/leu.2017.136 PMC5729333

[36] Piovan E, Yu J, Tosello V, Herranz D, Ambesi-Impiombato A, DaSilva AC, Sanchez-Martin M, Perez-Garcia A, Rigo I, Castillo M, et al. Direct Reversal of Glucocorticoid Resistance by AKT Inhibition in Acute Lymphoblastic Leukemia. Cancer Cell. 2013;24:766–76. 24291004 10.1016/j.ccr.2013.10.022 PMC3878658

[37] Meyer LK, Huang BJ, Delgado-Martin C, Roy RP, Hechmer A, Wandler AM, Vincent TL, Fortina P, Olshen AB, Wood BL, et al. Glucocorticoids Paradoxically Facilitate Steroid Resistance in T Cell Acute Lymphoblastic Leukemias and Thymocytes. J Clin Investig. 2020;130:863–76. 31687977 10.1172/JCI130189 PMC6994137

[38] Kośmider K, Karska K, Kozakiewicz A, Lejman M, Zawitkowska J. Overcoming Steroid Resistance in Pediatric Acute Lymphoblastic Leukemia-The State-of-the-Art Knowledge and Future Prospects. Int J Mol Sci. 2022;23(7):3795. 35409154 10.3390/ijms23073795 PMC8999045

[39] Roderick JE, Gallagher KM, Murphy LC, O’Connor KW, Tang K, Zhang B, Brehm MA, Greiner DL, Yu J, Zhu LJ, et al. Prostaglandin E2 Stimulates CAMP Signaling and Resensitizes Human Leukemia Cells to Glucocorticoid-Induced Cell Death. Blood. 2021;137:500–12. 10.1182/blood.2020005712. 33507291 10.1182/blood.2020005712 PMC7845005

[40] Li XJ, Luo XQ, Han BW, Duan FT, Wei PP, Chen YQ. MicroRNA-100/99a, Deregulated in Acute Lymphoblastic Leukaemia, Suppress Proliferation and Promote Apoptosis by Regulating the FKBP51 and IGF1R/MTOR Signalling Pathways. Br. J. Cancer. 2013;109:2189–98. 10.1038/bjc.2013.562. 24030073 10.1038/bjc.2013.562 PMC3798962

[41] Liang YN, Tang YL, Ke ZY, Chen YQ, Luo XQ, Zhang H, Huang LB. MiR-124 Contributes to Glucocorticoid Resistance in Acute Lymphoblastic Leukemia by Promoting Proliferation, Inhibiting Apoptosis and Targeting the Glucocorticoid Receptor. J Steroid Biochem Mol Biol. 2017;172:62–8. 10.1016/j.jsbmb.2017.05.014. 28578002 10.1016/j.jsbmb.2017.05.014

[42] Paugh SW, Bonten EJ, Savic D, Ramsey LB, Thierfelder WE, Gurung P, Malireddi RKS, Actis M, Mayasundari A, Min J, et al. NALP3 Inflammasome Upregulation and CASP1 Cleavage of the Glucocorticoid Receptor Cause Glucocorticoid Resistance in Leukemia Cells. Nat Genet. 2015;47:607–14. 25938942 10.1038/ng.3283 PMC4449308

[43] Burke MJ, Ziegler DS, Bautista Sirvent FJ, Attarbaschi A, Gore L, Locatelli F, O’Brien MM, Pauly M, Obreja M, Morris CL, et al. Phase 1b Study of Carfilzomib in Combination with Induction Chemotherapy in Children with Relapsed or Refractory Acute Lymphoblastic Leukemia (ALL). Blood. 2019;134:3873. 10.1182/blood-2019-127350. 10.1182/blood-2019-127350

[44] Jonas BA, Fisch SC, Rosenberg AS, Hoeg RT, Tuscano JM, Abedi M. Phase I Study of Escalating Doses of Carfilzomib with HyperCVAD in Patients with Newly Diagnosed Acute Lymphoblastic Leukemia. Am J Hematol. 2021;96:E114–7. 33476436 10.1002/ajh.26105

[45] Sarno J, Domizi P, Liu Y, et al. Dasatinib overcomes glucocorticoid resistance in B-cell acute lymphoblastic leukemia. Nat Commun. 2023;14:2935. 37217509 10.1038/s41467-023-38456-y PMC10203345

[46] Kaspers GJ, Veerman AJ, Pieters R, et al. In vitro cellular drug resistance and prognosis in newly diagnosed childhood acute lymphoblastic leukemia. Blood. 1997;90:2723–9. 9326239 10.1182/blood.V90.7.2723

[47] Den Boer ML, Harms DO, Pieters R, et al. Patient stratification based on prednisolone-vincristine-asparaginase resistance profiles in children with acute lymphoblastic leukemia. J Clin Oncol. 2003;21:3262–8. 12947061 10.1200/JCO.2003.11.031

[48] Hunger SP, Mullighan CG. Acute lymphoblastic leukemia in children. N Engl J Med. 2015;373:1541–52. 26465987 10.1056/NEJMra1400972

[49] Real PJ, Ferrando AA. NOTCH inhibition and glucocorticoid therapy in T-cell acute lymphoblastic leukemia. Leukemia. 2009;23:1374–7. 19357700 10.1038/leu.2009.75 PMC2814171

[50] Zweidler-McKay PA, Hogan MS, Jubran R, et al. Navigating your career path in pediatric hematology/oncology: On and off the beaten track. Pediatr Blood Cancer. 2016;63:1723–30. 27295503 10.1002/pbc.26094

[51] Delgado-Martin C, Meyer LK, Huang BJ, et al. JAK/STAT pathway inhibition overcomes IL7-induced glucocorticoid resistance in a subset of human T-cell acute lymphoblastic leukemias. Leukemia. 2017;31:2568–76. 28484265 10.1038/leu.2017.136 PMC5729333

[52] Meyer LK, Delgado-Martin C, Sharp PP, et al. Inhibition of the Sec61 translocon overcomes cytokine-induced glucocorticoid resistance in T-cell acute lymphoblastic leukaemia. Br J Haematol. 2022;198:137–41. 35434798 10.1111/bjh.18181 PMC9322670

[53] Pufall MA. Glucocorticoids and Cancer. Adv Exp Med Biol. 2015;872:315–33. 26216001 10.1007/978-1-4939-2895-8_14 PMC5546099

[54] Velentza L, Zaman F, Sävendahl L. Bone health in glucocorticoid-treated childhood acute lymphoblastic leukemia. Crit Rev Oncol Hematol. 2021 Dec;168:103492. 34655742 10.1016/j.critrevonc.2021.103492

[55] Mushtaq T, Ahmed SF. The impact of corticosteroids on growth and bone health. Arch Dis Child. 2002;87(2):93–6. 12138051 10.1136/adc.87.2.93 PMC1719206

[56] te Winkel ML, Pieters R, Hop WC, Roos JC, Bokkerink JP, Leeuw JA, et al. Bone mineral density at diagnosis determines fracture rate in children with acute lymphoblastic leukemia treated according to the DCOG-ALL9 protocol. Bone. 2014;59:223–8. 24287213 10.1016/j.bone.2013.11.017

[57] Rayar MS, Nayiager T, Webber CE, Barr RD, Athale UH. Predictors of bony morbidity in children with acute lymphoblastic leukemia. Pediatr Blood Cancer. 2012;59(1):77–82. 22190454 10.1002/pbc.24040

[58] Cummings EA, Ma J, Fernandez CV, Halton J, Alos N, Miettunen PM, et al. Incident vertebral fractures in children with leukemia during the four years following diagnosis. J Clin Endocrinol Metab. 2015;100(9):3408–17. 26171800 10.1210/JC.2015-2176 PMC4909472

[59] Mostoufi-Moab S, Brodsky J, Isaacoff EJ, Tsampalieros A, Ginsberg JP, Zemel B, et al. Longitudinal assessment of bone density and structure in childhood survivors of acute lymphoblastic leukemia without cranial radiation. J Clin Endocrinol Metab. 2012;97(10):3584–92. 22865901 10.1210/jc.2012-2393 PMC3674298

[60] Orgel E, Mueske NM, Wren TA, Gilsanz V, Butturini AM, Freyer DR, et al. Early injury to cortical and cancellous bone from induction chemotherapy for adolescents and young adults treated for acute lymphoblastic leukemia. Bone. 2016;85:131–7. 26851412 10.1016/j.bone.2016.01.027 PMC4795805

[61] Jarfelt M, Fors H, Lannering B, Bjarnason R. Bone mineral density and bone turnover in young adult survivors of childhood acute lymphoblastic leukaemia. Eur J Endocrinol. 2006;154(2):303–9. 16452545 10.1530/eje.1.02092

[62] van Atteveld JE, Pluijm SMF, Ness KK, Hudson MM, Chemaitilly W, Kaste SC, et al. Prediction of low and very low bone mineral density among adult survivors of childhood cancer. J Clin Oncol. 2019;37(25):2217–25. 31169453 10.1200/JCO.18.01917 PMC6804829

[63] Inaba H, Cao X, Han AQ, Panetta JC, Ness KK, Metzger ML, et al. Bone mineral density in children with acute lymphoblastic leukemia. Cancer. 2018;124(5):1025–35. 29266176 10.1002/cncr.31184 PMC5821586

[64] Ward LM, Ma J, Lang B, Ho J, Alos N, Matzinger MA, et al. Bone morbidity and recovery in children with acute lymphoblastic leukemia: results of a six-year prospective cohort study. J Bone Miner Res. 2018;33(8):1435–43. 29786884 10.1002/jbmr.3447

[65] Elmantaser M, Stewart G, Young D, Duncan R, Gibson B, Ahmed SF. Skeletal morbidity in children receiving chemotherapy for acute lymphoblastic leukaemia. Arch Dis Child. 2010;95(10):805–9. 20576660 10.1136/adc.2009.172528

[66] Mattano LA, Sather HN, Trigg ME, Nachman JB. Osteonecrosis as a complication of treating acute lymphoblastic leukemia in children: a report from the Children’s Cancer Group. J Clin Oncol. 2000;18(18):3262–3272 70. 10986059 10.1200/JCO.2000.18.18.3262

[67] Strauss AJ, Su JT, Dalton VM, Gelber RD, Sallan SE, Silverman LB. Bony morbidity in children treated for acute lymphoblastic leukemia. J Clin Oncol. 2001;19(12):3066–72. 11408503 10.1200/JCO.2001.19.12.3066

[68] Mattano LA, Devidas M, Nachman JB, et al. Children’s Oncology GroupEffect of alternate-week versus continuous dexamethasone scheduling on the risk of osteonecrosis in paediatric patients with acute lymphoblastic leukaemia: results from the CCG-1961 randomized cohort trial. Lancet Oncol. 2012;13(9):906–15. 22901620 10.1016/S1470-2045(12)70274-7 PMC3448283

[69] Hyakuna N, Shimomura Y, Watanabe A, et al. Japanese Childhood Cancer and Leukemia Study Group (JCCLSG)Assessment of corticosteroid-induced osteonecrosis in children undergoing chemotherapy for acute lymphoblastic leukemia: a report from the Japanese Childhood Cancer and Leukemia Study Group. J Pediatr Hematol/Oncol. 2014;36(1):22–9. 24136019 10.1097/MPH.0000000000000039 PMC3872830

[70] Burger B, Beier R, Zimmermann M, Beck JD, Reiter A, Schrappe M. Osteonecrosis: a treatment related toxicity in childhood acute lymphoblastic leukemia (ALL)--experiences from trial ALL-BFM 95. Pediatr Blood Cancer. 2005;44:220–5. 15514916 10.1002/pbc.20244

[71] Kadan-Lottick NS, Dinu I, Wasilewski-Masker K, et al. Osteonecrosis in adult survivors of childhood cancer: a report from the childhood cancer survivor study. J Clin Oncol. 2008;26:3038–45. 18565890 10.1200/JCO.2007.14.9088 PMC9478878

[72] Valtis YK, Stevenson KE, Place AE, Silverman LB, Vrooman LM, Gotti G, Brunner AM, Nauffal M, DeAngelo DJ, Luskin MR. Orthopedic toxicities among adolescents and young adults treated in DFCI ALL Consortium Trials. Blood Adv. 2022 Jan 11;6(1):72–81. 34610104 10.1182/bloodadvances.2021005278 PMC8753211

[73] Patel B, Richards SM, Rowe JM, Goldstone AH, Fielding AK. High incidence of avascular necrosis in adolescents with acute lymphoblastic leukaemia: a UKALL XII analysis. Leukemia. 2008;22(2):308–12. 17989709 10.1038/sj.leu.2405032

[74] Toft N, Birgens H, Abrahamsson J, et al. Toxicity profile and treatment delays in NOPHO ALL2008-comparing adults and children with Philadelphia chromosome-negative acute lymphoblastic leukemia. Eur J Haematol. 2016;96(2):160–9. 25867866 10.1111/ejh.12562

[75] Yao S, Zhu Q, Cole PD, et al. Genetic ancestry and skeletal toxicities among childhood acute lymphoblastic leukemia patients in the DFCI 05-001 cohort. Blood Adv. 2021;5(2):451–8. 33496737 10.1182/bloodadvances.2020003060 PMC7839368

[76] Hurwitz CA, Silverman LB, Schorin MA, et al. Substituting dexamethasone for prednisone complicates remission induction in children with acute lymphoblastic leukemia. Cancer. 2000;88:1964–9. 10760775 10.1002/(SICI)1097-0142(20000415)88:8<1964::AID-CNCR27>3.0.CO;2-1

[77] Gregoriou K, Craigie I, Gibson B, Mason A, Shaikh MG. Risk factors and management of corticosteroid-induced hyperglycaemia in paediatric acute lymphoblastic leukaemia. Pediatr Blood Cancer. 2020;67:e28085. 31736211 10.1002/pbc.28085

[78] Beaudry J, Riddell M. Effects of glucocorticoids and exercise on pancreatic β-cell function and diabetes development. Diabetes Metab Res Rev. 2012;28(7):560–73. 22556149 10.1002/dmrr.2310

[79] Lowas S, Malempati S, Marks D. Body mass index predicts insulin resistance in survivors of pediatric acute lymphoblastic leukemia. Pediatr Blood Cancer. 2009;53(1):58–63. 19340854 10.1002/pbc.21993 PMC3804011

[80] Weiser M, Cabanillas M, Konopleva M, et al. Relation between the duration of remission and hyperglycemia during induction chemotherapy for acute lymphocytic leukemia with a hyperfractionated cyclophosphamide, vincristine, doxorubicin, and dexamethasone/methotrexate-cytarabine regimen. Cancer. 2004;100(6):1179–85. 15022284 10.1002/cncr.20071

[81] Lowas S, Marks D, Malempati S. Prevalence of transient hyperglycemia during induction chemotherapy for pediatric acute lymphoblastic leukemia. Pediatr Blood Cancer. 2009;52(7):814–8. 19260096 10.1002/pbc.21980

[82] Koltin D, Sung L, Naqvi A, Urbach S. Medication induced diabetes during induction in pediatric acute lymphoblastic leukemia: prevalence, risk factors and characteristics. Support Care Cancer. 2011;20(9):2009–15. 22065148 10.1007/s00520-011-1307-5

[83] Gatzioura I, Papakonstantinou E, Dimitriadou M, et al. Glucose levels before the onset of asparaginase predicts transient hyperglycemia in children with acute lymphoblastic leukemia. Pediatr Blood Cancer. 2016;63(7):1181–4. 27062362 10.1002/pbc.25956

[84] Dare J, Moppett J, Shield J, Hunt L, Stevens M. The impact of hyperglycemia on risk of infection and early death during induction therapy for acute lymphoblastic leukemia (ALL). Pediatr Blood Cancer. 2013;60(12):E157–9. 23868820 10.1002/pbc.24689

[85] Roberson J, Raju S, Shelso J, Pui C, Howard S. Diabetic ketoacidosis during therapy for pediatric acute lymphoblastic leukemia. Pediatr Blood Cancer. 2008;50(6):1207–12. 18266226 10.1002/pbc.21505

[86] Dropcho EJ, Soong SJ. Steroid-induced weakness in patients with primary brain tumors. Neurology. 1991;41:1235–9. 1866012 10.1212/WNL.41.8.1235

[87] Wolkowitz OM. Prospective controlled studies of the behavioral and biological effects of exogenous corticosteroids. Psychoneuroendocrinology. 1994;19:233–55. 7515507 10.1016/0306-4530(94)90064-7

[88] Danilczuk Z, Ossowska G, Lupina T, Cieslik K, Zebrowska-Lupina I. Effect of NMDA receptor antagonists on behavioral impairment induced by chronic treatment with dexamethasone. Pharmacol Rep. 2005;57:47–54. 15849376

[89] Waber DP, Carpentieri SC, Klar N, et al. Cognitive sequelae in children treated for acute lymphoblastic leukemia with dexamethasone or prednisone. J Pediatr Hematol Oncol. 2000;22:206–13. 10864051 10.1097/00043426-200005000-00004

[90] Kadan-Lottick NS, Brouwers P, Breiger D, et al. A comparison of neurocognitive functioning in children previously randomized to dexamethasone or prednisone in the treatment of childhood acute lymphoblastic leukemia. Blood. 2009;114:1746. 19546477 10.1182/blood-2008-12-186502 PMC2738566

[91] Rangel-Patiño J, Lee-Tsai YL, Urbalejo-Ceniceros VI, Luna-Perez MEM, et al. A modified CALGB 10403 in adolescents and young adults with acute lymphoblastic leukemia in Central America. Blood Adv. 2023;7(18):5202–9. 37307212 10.1182/bloodadvances.2023009754 PMC10500455

[92] Aumann S, Shaulov A, Haran A, Gross Even-Zohar N, Vainstein V, Nachmias B. The Emerging Role of Venetoclax-Based Treatments in Acute Lymphoblastic Leukemia. Int J Mol Sci. 2022;23(18):10957. 36142863 10.3390/ijms231810957 PMC9504828

[93] Peirs S, Matthijssens F, Goossens S, Van De Walle I, Ruggero K, De Bock CE, et al. ABT-199 mediated inhibition of BCL-2 as a novel therapeutic strategy in T-cell acute lymphoblastic leukemia. Blood. 2014;124:3738–47. 25301704 10.1182/blood-2014-05-574566

[94] Rahmat LT, Nguyen A, Abdulhaq H, Prakash S, Logan AC, Mannis GN. Venetoclax in combination with decitabine for relapsed T-cell acute lymphoblastic leukemia after allogeneic hematopoietic cell transplant. Case Rep Hematol. 2018;2018:6092646. 30225152 10.1155/2018/6092646 PMC6129347

[95] Farhadfar N, Li Y, May WS, Adams CB. Venetoclax and decitabine for treatment of relapsed T-cell acute lymphoblastic leukemia: A case report and review of literature. Hematol Oncol Stem Cell Ther. 2021;14:246–51. 32199933 10.1016/j.hemonc.2019.10.002

[96] Ni Chonghaile T, Roderick JE, Glenfield C, Ryan J, Sallan SE, Silverman LB, et al. Maturation stage of T-cell acute lymphoblastic leukemia determines BCL-2 versus BCL-XL dependence and sensitivity to ABT-199. Cancer Discov. 2014;4:1074–87. 24994123 10.1158/2159-8290.CD-14-0353 PMC4154982

[97] Hohtari H, Kankainen M, Adnan-Awad S, Yadav B, Potdar S, Ianevski A, et al. Targeting apoptosis pathways with BCL2 and MDM2 inhibitors in adult B-cell acute lymphoblastic leukemia. HemaSphere. 2022;6:e701. 35233509 10.1097/HS9.0000000000000701 PMC8878725

[98] Zhang Y, Qian JJ, Shen YJ, Hang SJ, Jin J, Zhu HH. The first report of complete remission following treatment with venetoclax plus prednisone in elderly patients with Philadelphia chromosome-negative acute lymphoblastic leukemia. Ann Hematol. 2022;101:1141–4. 34661714 10.1007/s00277-021-04699-2

[99] Katsuya H, Cook LBM, Rowan AG, Satou Y, Taylor GP, Bangham CRM. Phosphatidylinositol 3-Kinase-δ (PI3K-δ) Is a Potential Therapeutic Target in Adult T-Cell Leukemia-Lymphoma. Biomark Res. 2018;6:24. 10.1186/s40364-018-0138-7. 30034808 10.1186/s40364-018-0138-7 PMC6052569

[100] Wandler AM, Huang BJ, Craig JW, Hayes K, Yan H, Meyer LK, Scacchetti A, et al. Loss of Glucocorticoid Receptor Expression Mediates in Vivo Dexamethasone Resistance in T-Cell Acute Lymphoblastic Leukemia. Leukemia. 2020;34:2025–37. 32066867 10.1038/s41375-020-0748-6 PMC7440098

[101] Bressanin D, Evangelisti C, Ricci F, Tabellini G, Chiarini F, Tazzari PL, Melchionda F, Buontempo F, Pagliaro P, Pession A, et al. Harnessing the PI3K/Akt/MTOR Pathway in T-Cell Acute Lymphoblastic Leukemia: Eliminating Activity by Targeting at Different Levels. Oncotarget. 2012;3:811–23. 22885370 10.18632/oncotarget.579 PMC3478458

[102] Ksionda O, Mues M, Wandler AM, Donker L, Tenhagen M, Jun J, Ducker GS, Matlawska-Wasowska K, Shannon K, Shokat KM, et al. Comprehensive Analysis of T Cell Leukemia Signals Reveals Heterogeneity in the PI3 Kinase-Akt Pathway and Limitations of PI3 Kinase Inhibitors as Monotherapy. PLoS ONE. 2018;13:e0193849. 29799846 10.1371/journal.pone.0193849 PMC5969748

[103] Dail M, Wong J, Lawrence J, O’Connor D, Nakitandwe J, Chen SC, Xu J, Lee LB, Akagi K, Li Q, et al. Loss of Oncogenic Notch1 with Resistance to a PI3K Inhibitor in T-Cell Leukaemia. Nature. 2014;513:512–6. 25043004 10.1038/nature13495 PMC4213126

[104] Pereira JKN, Machado-Neto JA, Lopes MR, Morini BC, Traina F, Costa FF, Saad STO, Favaro P. Molecular Effects of the Phosphatidylinositol-3-Kinase Inhibitor NVP-BKM120 on T and B-Cell Acute Lymphoblastic Leukaemia. Eur J Cancer. 2015;51:2076–85. 26238016 10.1016/j.ejca.2015.07.018

[105] Lonetti A, Antunes IL, Chiarini F, Orsini E, Buontempo F, Ricci F, Tazzari PL, Pagliaro P, Melchionda F, Pession A, et al. Activity of the Pan-Class I Phosphoinositide 3-Kinase Inhibitor NVP-BKM120 in T-Cell Acute Lymphoblastic Leukemia. Leukemia. 2014;28:1196–206. 24310736 10.1038/leu.2013.369

[106] Delgado-Martin C, Meyer LK, Huang BJ, Shimano KA, Zinter MS, Nguyen JV, Smith GA, Taunton J, Winter SS, Roderick JR, et al. JAK/STAT Pathway Inhibition Overcomes IL7-Induced Glucocorticoid Resistance in a Subset of Human T-Cell Acute Lymphoblastic Leukemias. Leukemia. 2017;31:2568–76. 28484265 10.1038/leu.2017.136 PMC5729333

[107] Böhm JW, Sia KCS, Jones C, Evans K, Mariana A, Pang I, Failes T, Zhong L, Mayoh C, Landman R, et al. Combination Efficacy of Ruxolitinib with Standard-of-Care Drugs in CRLF2-Rearranged Ph-like Acute Lymphoblastic Leukemia. Leukemia. 2021;35:3101–12. 33895784 10.1038/s41375-021-01248-8

[108] Van der Zwet JCG, Buijs-Gladdines JGCAM, Cordo V, Debets DO, Smits WK, Chen Z, Dylus J, Zaman GJR, Altelaar M, Oshima K, et al. MAPK-ERK Is a Central Pathway in T-Cell Acute Lymphoblastic Leukemia That Drives Steroid Resistance. Leukemia. 2021;35:3394–405. 34007050 10.1038/s41375-021-01291-5

[109] Degryse S, de Bock CE, Demeyer S, Govaerts I, Bornschein S, Verbeke D, Jacobs K, Binos S, Skerrett-Byrne DA, Murray HC, et al. Mutant JAK3 Phosphoproteomic Profiling Predicts Synergism between JAK3 Inhibitors and MEK/BCL2 Inhibitors for the Treatment of T-Cell Acute Lymphoblastic Leukemia. Leukemia. 2018;32:788–800. 28852199 10.1038/leu.2017.276 PMC5843905

[110] Irving J, Matheson E, Minto L, Blair H, Case M, Halsey C, Swidenbank I, Ponthan F, Kirschner-Schwabe R, Groeneveld-Krentz S, et al. Ras Pathway Mutations Are Prevalent in Relapsed Childhood Acute Lymphoblastic Leukemia and Confer Sensitivity to MEK Inhibition. Blood. 2014;124:3420–30. 25253770 10.1182/blood-2014-04-531871 PMC4246039

[111] Polak A, Kiliszek P, Sewastianik T, Szydłowski M, Jabłońska E, Białopiotrowicz E, Górniak P, Markowicz S, Nowak E, Grygorowicz MA, et al. MEK Inhibition Sensitizes Precursor B-Cell Acute Lymphoblastic Leukemia (B-ALL) Cells to Dexamethasone through Modulation of MTOR Activity and Stimulation of Autophagy. PLoS ONE. 2016;11:e0155893. 27196001 10.1371/journal.pone.0155893 PMC4872998

[112] Kerstjens M, Driessen EMC, Willekes M, Pinhanços SS, Schneider P, Pieters R, Stam RW. MEK Inhibition Is a Promising Therapeutic Strategy for MLL-Rearranged Infant Acute Lymphoblastic Leukemia Patients Carrying RAS Mutations. Oncotarget. 2017;8:14835–46. 27588400 10.18632/oncotarget.11730 PMC5362448

[113] Esbenshade AJ, Simmons JH, Koyama T, Lindell RB, Friedman DL. Obesity and insulin resistance in pediatric acute lymphoblastic leukemia worsens during maintenance therapy. Pediatr Blood Cancer. 2013 Aug;60(8):1287–91. 23444342 10.1002/pbc.24489 PMC3881979

[114] Garmey EG, Liu Q, Sklar CA, et al. Longitudinal changes in obesity and body mass index among adult survivors of childhood acute lymphoblastic leukemia: A report from the childhood cancer survivor study. J Clin Oncol. 2008;26:4639–45. 18824710 10.1200/JCO.2008.16.3527 PMC2653124

[115] Orgel E, Tucci J, Alhushki W, Malvar J, Sposto R, Fu CH, Freyer DR, Abdel-Azim H, Mittelman SD. Obesity is associated with residual leukemia following induction therapy for childhood B-precursor acute lymphoblastic leukemia. Blood. 2014;124(26):3932–8. 25349177 10.1182/blood-2014-08-595389

[116] Wilding JPH, Batterham RL, Calanna S, Davies M, Van Gaal LF, Lingvay I, McGowan BM, Rosenstock J, Tran MTD, Wadden TA, Wharton S, Yokote K, Zeuthen N. Kushner RF; STEP 1 Study Group. Once-Weekly Semaglutide in Adults with Overweight or Obesity. N Engl J Med. 2021;384(11):989–1002. 33567185 10.1056/NEJMoa2032183

[117] Popoviciu MS, Păduraru L, Yahya G, Metwally K, Cavalu S. Emerging Role of GLP-1 Agonists in Obesity: A Comprehensive Review of Randomised Controlled Trials. Int J Mol Sci. 2023 Jun 21;24(13):10449. 37445623 10.3390/ijms241310449 PMC10341852

